# Supplementary material for: A Tale of Two Families: Whole Genome and Segmental Duplications Underlie Glutamine Synthetase and Phosphoenolpyruvate Carboxylase Diversity in Narrow-Leafed Lupin (Lupinus angustifolius L.)
Source: Int J Mol Sci. 2020 Apr 8;21(7):2580. doi: 10.3390/ijms21072580 (PMC7177731; doi:10.3390/ijms21072580)
Supplement: Supplementary file 1 [file ijms-21-02580-s001.zip › Supplementary files/Supplementary file 10.docx]

**Title:** Tale of two families – whole genome and segmental duplications underlie glutamine synthetases and phosphoenolpyruvate carboxylases diversity in narrow-leafed lupin

**Authors:** Katarzyna B. Czyż, Michał Książkiewicz, Grzegorz Koczyk, Anna Szczepaniak, Jan Podkowiński, Barbara Naganowska

**Journal:** International Journal of Molecular Sciences

**Supplementary file 10.** List of single-ortholog loci used in species tree reconstruction and optimal models found by IQTREE model testing.

| **#** | ***A. thaliana* locus** | **Selected model** | **Description** |
| --- | --- | --- | --- |
| 1 | AT1G13120 | SCHN05+F+G4 | nucleoporin GLE1-like protein |
| 2 | AT1G18335 | SCHN05+G4 | Acyl-CoA N-acyltransferases (NAT) superfamily protein |
| 3 | AT2G19385 | SCHN05+F+G4 | Zinc ion binding protein |
| 4 | AT2G25605 | SCHN05+G4 | DNA-directed RNA polymerase subunit beta |
| 5 | AT2G30320 | SCHN05+G4 | Putative tRNA pseudouridine synthase |
| 6 | AT2G38780 | SCHN05+F+G4 | Cytochrome C oxidase subunit |
| 7 | AT3G05070 | SCHN05+G4 | Cwf18 pre-mRNA splicing factor |
| 8 | AT3G48210 | MGK+F3X4+G4 | Kinetochore protein |
| 9 | AT3G49080 | SCHN05+G4 | Ribosomal protein S5 domain 2-like superfamily protein |
| 10 | AT3G54630 | SCHN05+F+G4 | Kinetochore protein T14E10_200 |
| 11 | AT3G55340 | SCHN05+F+G4 | Phragmoplastin interacting protein 1 |
| 12 | AT3G60660 | SCHN05+G4 | Spindle and kinetochore-associated protein 1 homolog |
| 13 | AT4G01880 | SCHN05+G4 | Uncharacterized protein T7B11.14, uncharacterised methyltransferase |
| 14 | AT4G04870 | SCHN05+G4 | cardiolipin synthase |
| 15 | AT4G12740 | SCHN05+G4 | HhH-GPD base excision DNA repair family protein |
| 16 | AT4G13670 | SCHN05+G4 | plastid transcriptionally active 5 (PTAC5), protein disulfide isomerase |
| 17 | AT4G26980 | SCHN05+G4 | RNI-like superfamily protein (CRK18) |
| 18 | AT4G28020 | SCHN05+G4 | tRNA-thr(GGU) m(6)t(6)A37 methyltransferase |
| 19 | AT4G32260 | SCHN05+G4 | ATPase, F0 complex, subunit B/B', chloroplast |
| 20 | AT4G35760 | SCHN05+G4 | NAD(P)H-dependent naphtoquinone reductase, thylakoid-associated disulfide bond oxidoreductase (LTO1) |
| 21 | AT4G38020 | SCHN05+G4 | tRNA/rRNA methyltransferase (SpoU) family protein |
| 22 | AT5G12220 | MGK+F3X4+I+G4 | las1-like family protein |
| 23 | AT5G16630 | SCHN05+F+G4 | RAD4 DNA repair family protein |
| 24 | AT5G20600 | SCHN05+F+G4 | ribosomal RNA processing-like protein |
| 25 | AT5G30495 | SCHN05+G4 | Fcf2 pre-rRNA processing protein |
| 26 | AT5G46840 | SCHN05+G4 | RNA-binding (RRM/RBD/RNP motifs) family protein |
| 27 | AT5G49010 | SCHN05+G4 | DNA replication protein-like protein involved in initiation of replication (SLD5) |
| 28 | AT5G58220 | SCHN05+G4 | Transthyretin-like S-allantoin synthase (ALNS) |
| 29 | AT5G59610 | SCHN05+G4 | Chaperone DnaJ-domain superfamily protein |
