# Supplementary material for: A Tale of Two Families: Whole Genome and Segmental Duplications Underlie Glutamine Synthetase and Phosphoenolpyruvate Carboxylase Diversity in Narrow-Leafed Lupin (Lupinus angustifolius L.)
Source: Int J Mol Sci. 2020 Apr 8;21(7):2580. doi: 10.3390/ijms21072580 (PMC7177731; doi:10.3390/ijms21072580)
Supplement: Supplementary file 1 [file ijms-21-02580-s001.zip › Supplementary files/Supplementary file 11.docx]

**Title:** Tale of two families – whole genome and segmental duplications underlie glutamine synthetases and phosphoenolpyruvate carboxylases diversity in narrow-leafed lupin

**Authors:** Katarzyna B. Czyż, Michał Książkiewicz, Grzegorz Koczyk, Anna Szczepaniak, Jan Podkowiński, Barbara Naganowska

**Journal:** International Journal of Molecular Sciences

**Supplematary file 11.** Fragment/outlier loci discarded doing gap profile (OD-seq) inspection of protein alignments. Legume sequences highlighted with bold font.

| **Group** | **Outliers** |
| --- | --- |
| **GS** | brassica_rapa__gene:Bra032257 |
|  | capsella_grandiflora__Cagra.5084s0001.v1.1 |
|  | carica_papaya__evm.TU.supercontig_116.73 |
|  | carica_papaya__evm.TU.supercontig_16.50 |
|  | **glycine_max__gene:GLYMA15G21061** |
|  | **lotus_japonicus__Lj6g3v0410480** |
|  | **lotus_japonicus__Lj6g3v0410490** |
|  | musa_acuminata__gene:GSMUA_Achr3G14160_001 |
|  | populus_trichocarpa__gene:POPTR_0005s14310 |
|  | populus_trichocarpa__gene:POPTR_0007s07960 |
|  | populus_trichocarpa__gene:POPTR_0017s02300 |
|  | populus_trichocarpa__gene:POPTR_0154s00240 |
|  | spirodela_polyrhiza__Spipo16G0030500.v2 |
| **PEPC** | **arachis_ipaensis__gene36286** |
|  | carica_papaya__evm.TU.supercontig_1.237 |
|  | carica_papaya__evm.TU.supercontig_97.104 |
|  | chlamydomonas_reinhardtii__gene:CHLREDRAFT_80312 |
|  | **glycine_max__gene:GLYMA02G14495** |
|  | hordeum_vulgare__gene:MLOC_39281 |
|  | **lotus_japonicus__Lj0g3v0198289** |
|  | **lotus_japonicus__Lj5g3v1792310** |
|  | malus_domestica__MDP0000462196.GDRv1.0 |
|  | **medicago_truncatula__gene:MTR_1g009850** |
|  | prunus_persica__gene:PRUPE_ppa002020mg |
|  | ricinus_communis__29634.t000111.TIGRR0.1 |
|  | **trifolium_pratense__gene:Tp57577_TGAC_v2_gene28308** |
|  | volvox_carteri__Vocar.0018s0035.v2.1 |
