# Supplementary material for: A Tale of Two Families: Whole Genome and Segmental Duplications Underlie Glutamine Synthetase and Phosphoenolpyruvate Carboxylase Diversity in Narrow-Leafed Lupin (Lupinus angustifolius L.)
Source: Int J Mol Sci. 2020 Apr 8;21(7):2580. doi: 10.3390/ijms21072580 (PMC7177731; doi:10.3390/ijms21072580)
Supplement: Supplementary file 1 [file ijms-21-02580-s001.zip › Supplementary files/Supplementary file 2.docx]

**Title:** Tale of two families – whole genome and segmental duplications underlie glutamine synthetases and phosphoenolpyruvate carboxylases diversity in narrow-leafed lupin

**Authors:** Katarzyna B. Czyż, Michał Książkiewicz, Grzegorz Koczyk, Anna Szczepaniak, Jan Podkowiński, Barbara Naganowska

**Journal:** International Journal of Molecular Sciences

**Supplematary file 2.** Listing of reference genomes analysed and corresponding database sources.

| **Subset** | **Order** | **Family** | | **Species** | **Source database** |
| --- | --- | --- | --- | --- | --- |
| **red algae** | *Cyanidiales* | *Cyanidiaceae* | | *Cyanidioschyzon merolae strain 10D* | Ensembl |
| **green algae** | *Chlamydomonadales* | *Chlamydomonadaceae* | | *Chlamydomonas reinhardtii* | Ensembl |
|  |  | *Volvocaceae* | | *Volvox carteri* | Phytozome |
|  | *Chlorococcales* | *Coccomyxaceae* | | *Coccomyxa subellipsoidea* | Phytozome |
| **mosses** | *Funariales* | *Funariaceae* | | *Physcomitrella patens* | Ensembl |
| **lycopods** | *Selaginellales* | *Selaginellaceae* | | *Selaginella moellendorffii* | Ensembl |
| **early-diverging angiosperms** | *Amborellales* | *Amborellaceae* | | *Amborella trichopoda* | Ensembl |
| **monocots** | *Alismatales* | *Araceae* | | *Spirodela polyrhiza* | Phytozome |
|  | *Alismatales* | *Zosteraceae* | | *Zostera marina* | Phytozome |
|  | *Poales* | *Bromeliaceae* | | *Ananas comosus* | Phytozome |
|  |  | *Poaceae* | | *Brachypodium distachyon* | Ensembl |
|  |  |  |  | *Hordeum vulgare subsp. vulgare* | Ensembl |
|  |  |  |  | *Oryza sativa Japonica Group* | Ensembl |
|  |  |  |  | *Setaria italica* | Ensembl |
|  |  |  |  | *Sorghum bicolor* | Ensembl |
|  |  |  |  | *Zea mays* | Ensembl |
|  | *Zingiberales* | *Musaceae* | | *Musa acuminata subsp. malaccensis* | Ensembl |
| **superasterids** | *Caryophyllales* | *Amaranthaceae* | | *Amaranthus hypochondriacus* | Phytozome |
|  |  | *Chenopodiaceae* | | *Beta vulgaris subsp. vulgaris* | Ensembl |
| **asterids** | *Lamiales* | *Phrymaceae* | | *Erythranthe guttata* | Phytozome |
|  | *Solanales* | *Solanaceae* | | *Solanum lycopersicum* | Ensembl |
|  |  |  |  | *Solanum tuberosum* | Ensembl |
| **rosids** | *Brassicales* | *Brassicaceae* | | *Arabidopsis thaliana* | Ensembl |
|  |  |  |  | *Brassica rapa subsp. pekinensis* | Ensembl |
|  |  |  |  | *Capsella grandiflora* | Phytozome |
|  |  | *Caricaceae* | | *Carica papaya* | Phytozome |
|  | *Cucurbitales* | *Cucurbitaceae* | | *Cucumis sativus* | Phytozome |
|  | *Malpighiales* | *Euphorbiaceae* | | *Ricinus communis* | Phytozome |
|  |  | *Salicaceae* | | *Populus trichocarpa* | Ensembl |
|  |  |  |  | *Salix purpurea* | Phytozome |
|  | *Malvales* | *Malvaceae* | | *Theobroma cacao* | Ensembl |
|  | *Rosales* | *Rosaceae* | | *Fragaria vesca* | Phytozome |
|  |  |  |  | *Malus domestica* | Phytozome |
|  |  |  |  | *Prunus persica* | Ensembl |
|  | *Sapindales* | *Rutaceae* | | *Citrus sinensis* | Phytozome |
|  | *Vitales* | *Vitaceae* | | *Vitis vinifera* | Ensembl |
| **legumes** | *Fabales* | *Fabaceae* | dalbergioids | *Arachis ipaensis* | NCBI/RefSeq |
|  |  |  | genistoids | *Lupinus angustifolius* | NCBI/RefSeq |
|  |  |  | IRLC | *Cicer arietinum* | NCBI/RefSeq |
|  |  |  |  | *Medicago truncatula* | Ensembl |
|  |  |  |  | *Trifolium pratense* | Ensembl |
|  |  |  | milletioids | *Cajanus cajan* | NCBI/RefSeq |
|  |  |  |  | *Glycine max* | Ensembl |
|  |  |  |  | *Phaseolus vulgaris* | Phytozome |
|  |  |  |  | *Vigna radiata* | NCBI/RefSeq |
|  |  |  | robinioids | *Lotus japonicus* | Phytozome??? |
