# Supplementary material for: A Tale of Two Families: Whole Genome and Segmental Duplications Underlie Glutamine Synthetase and Phosphoenolpyruvate Carboxylase Diversity in Narrow-Leafed Lupin (Lupinus angustifolius L.)
Source: Int J Mol Sci. 2020 Apr 8;21(7):2580. doi: 10.3390/ijms21072580 (PMC7177731; doi:10.3390/ijms21072580)

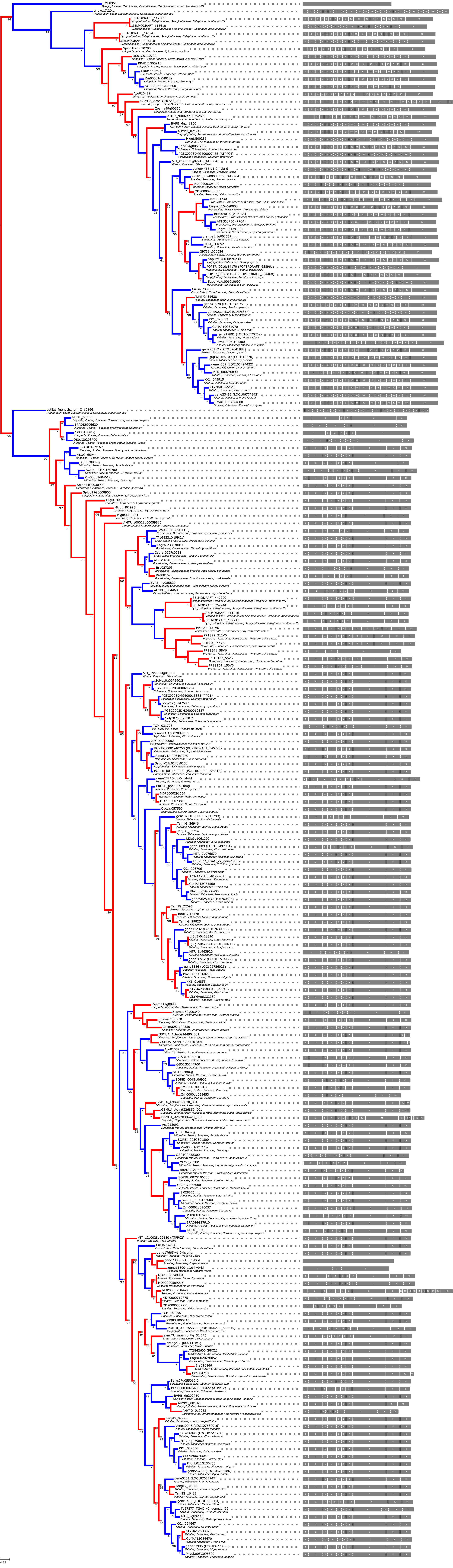

Supplement: Supplementary file 1 [file Supplementary file 6.pdf › Supplementary files/Supplementary file 6.pdf]
